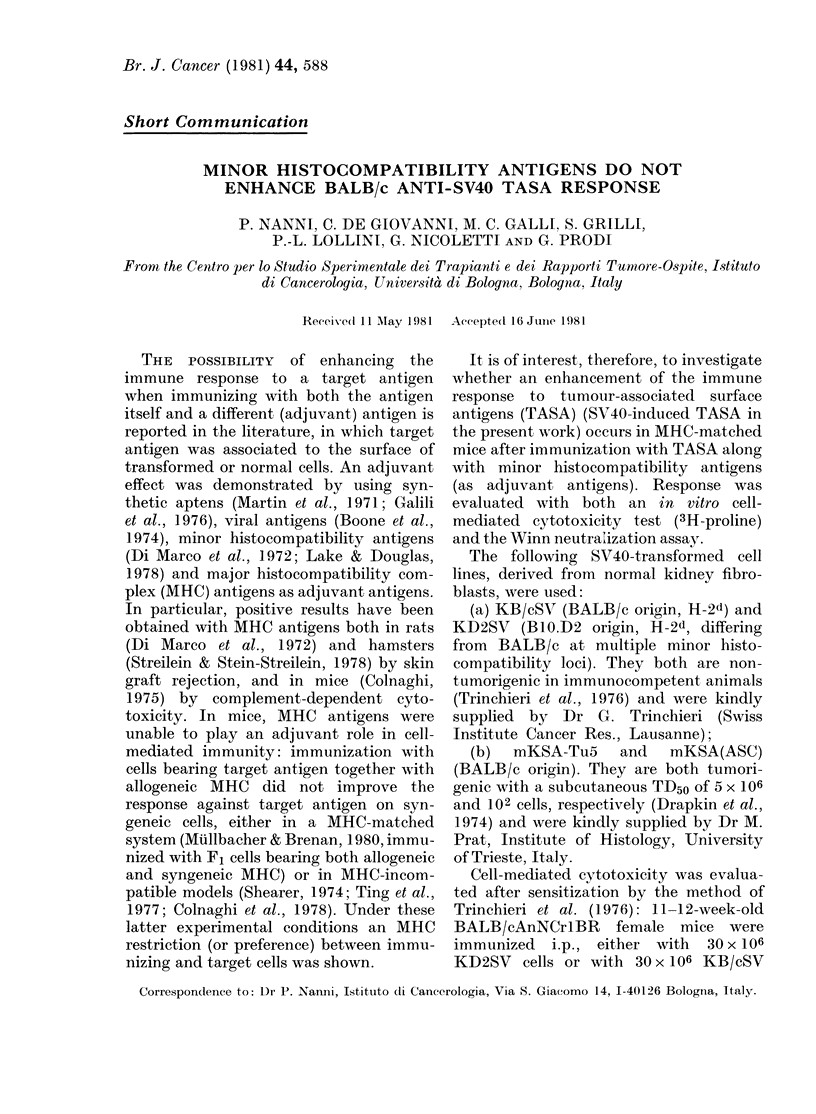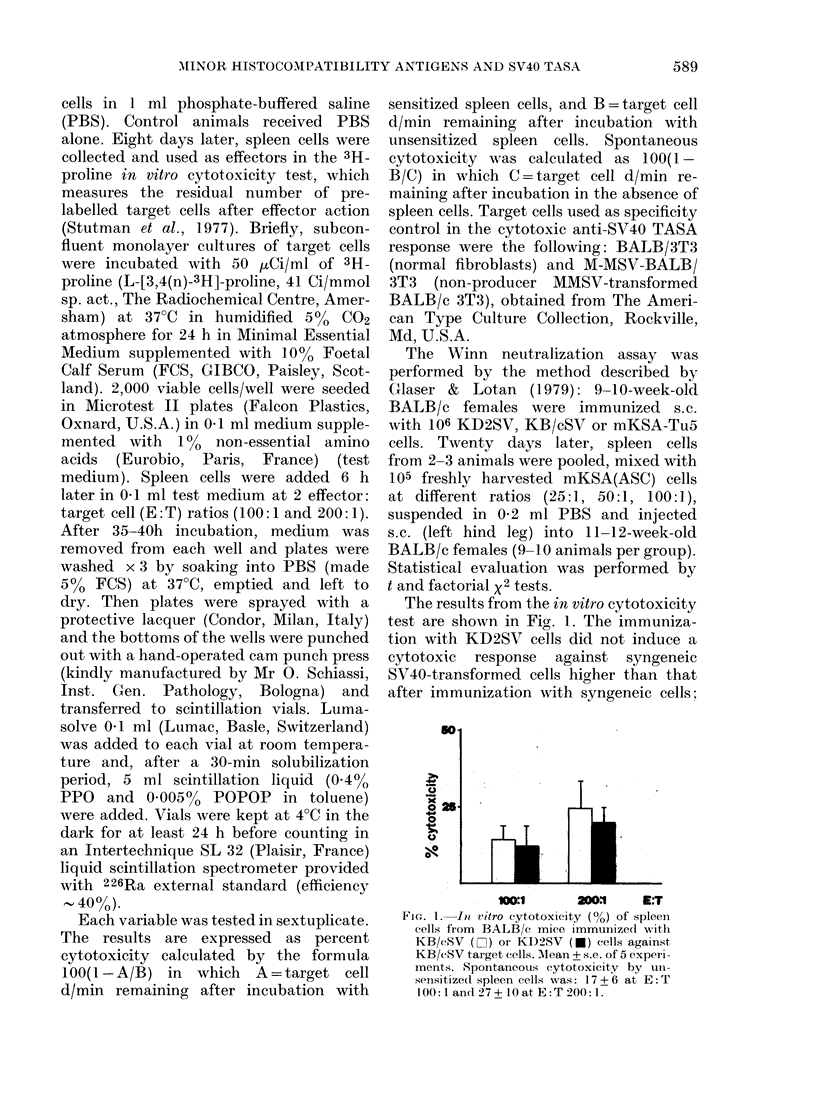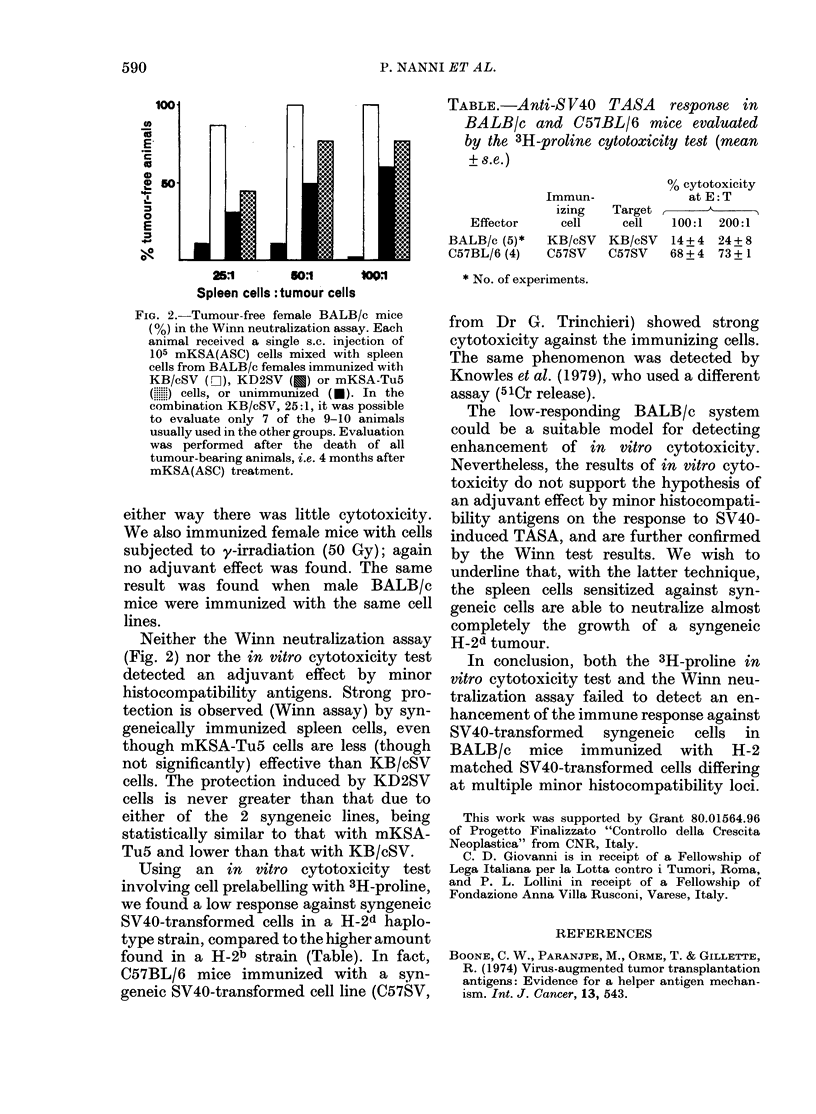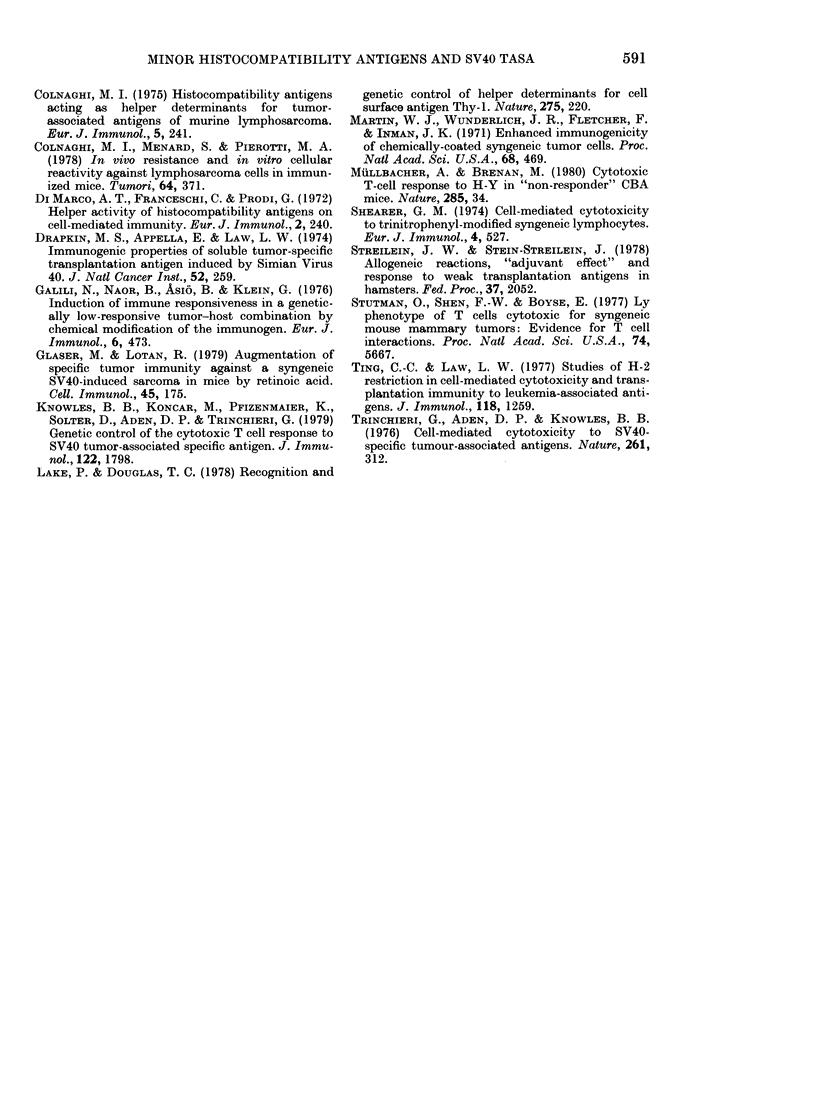# Minor histocompatibility antigens do not enhance BALB/c anti-SV40 TASA response.

**DOI:** 10.1038/bjc.1981.231

**Published:** 1981-10

**Authors:** P. Nanni, C. de Giovanni, M. C. Galli, S. Grilli, P. L. Lollini, G. Nicoletti, G. Prodi


*Br. J. Cancer* (1981) **44**, 588

***Short Communication***

### MINOR HISTOCOMPATIBILITY ANTIGENS DO NOT ENHANCE BALB/c ANTI-SV40 TASA RESPONSE

P. NANNI, C. DE GIOVANNI, M. C. GALLI, S. GRILLI, P.-L. LOLLINI, G. NICOLETTI AND G. PRODI

*From the Centro per lo Studio Sperimentale dei Trapianti e dei Rapporti Tumore-Ospite, Istituto di Cancerologia, Università di Bologna, Bologna, Italy*

Received 11 May 1981 Accepted 16 June 1981

THE POSSIBILITY of enhancing the immune response to a target antigen when immunizing with both the antigen itself and a different (adjuvant) antigen is reported in the literature, in which target antigen was associated to the surface of transformed or normal cells. An adjuvant effect was demonstrated by using synthetic aptens (Martin *et al.*, 1971; Galili *et al.*, 1976), viral antigens (Boone *et al.*, 1974), minor histocompatibility antigens (Di Marco *et al.*, 1972; Lake & Douglas, 1978) and major histocompatibility complex (MHC) antigens as adjuvant antigens. In particular, positive results have been obtained with MHC antigens both in rats (Di Marco *et al.*, 1972) and hamsters (Streilein & Stein-Streilein, 1978) by skin graft rejection, and in mice (Colnaghi, 1975) by complement-dependent cytotoxicity. In mice, MHC antigens were unable to play an adjuvant role in cell-mediated immunity: immunization with cells bearing target antigen together with allogeneic MHC did not improve the response against target antigen on syngeneic cells, either in a MHC-matched system (Müllbacher & Brenan, 1980, immunized with $F_1$ cells bearing both allogeneic and syngeneic MHC) or in MHC-incompatible models (Shearer, 1974; Ting *et al.*, 1977; Colnaghi *et al.*, 1978). Under these latter experimental conditions an MHC restriction (or preference) between immunizing and target cells was shown.

It is of interest, therefore, to investigate whether an enhancement of the immune response to tumour-associated surface antigens (TASA) (SV40-induced TASA in the present work) occurs in MHC-matched mice after immunization with TASA along with minor histocompatibility antigens (as adjuvant antigens). Response was evaluated with both an *in vitro* cell-mediated cytotoxicity test ($^3$H-proline) and the Winn neutralization assay.

The following SV40-transformed cell lines, derived from normal kidney fibroblasts, were used:

(a) KB/cSV (BALB/c origin, H-2$^d$) and KD2SV (B10.D2 origin, H-2$^d$, differing from BALB/c at multiple minor histocompatibility loci). They both are non-tumorigenic in immunocompetent animals (Trinchieri *et al.*, 1976) and were kindly supplied by Dr G. Trinchieri (Swiss Institute Cancer Res., Lausanne);

(b) mKSA-Tu5 and mKSA(ASC) (BALB/c origin). They are both tumorigenic with a subcutaneous $TD_{50}$ of $5 \times 10^6$ and $10^2$ cells, respectively (Drapkin *et al.*, 1974) and were kindly supplied by Dr M. Prat, Institute of Histology, University of Trieste, Italy.

Cell-mediated cytotoxicity was evaluated after sensitization by the method of Trinchieri *et al.* (1976): 11–12-week-old BALB/cAnNCrlBR female mice were immunized i.p., either with $30 \times 10^6$ KD2SV cells or with $30 \times 10^6$ KB/cSV

Correspondence to: Dr P. Nanni, Istituto di Cancerologia, Via S. Giacomo 14, I-40126 Bologna, Italy.

cells in 1 ml phosphate-buffered saline (PBS). Control animals received PBS alone. Eight days later, spleen cells were collected and used as effectors in the $^{3}$H-proline *in vitro* cytotoxicity test, which measures the residual number of pre-labelled target cells after effector action (Stutman *et al.*, 1977). Briefly, subconfluent monolayer cultures of target cells were incubated with 50 $\mu$Ci/ml of $^{3}$H-proline (L-[3,4(n)-$^{3}$H]-proline, 41 Ci/mmol sp. act., The Radiochemical Centre, Amersham) at 37°C in humidified 5% $CO_2$ atmosphere for 24 h in Minimal Essential Medium supplemented with 10% Foetal Calf Serum (FCS, GIBCO, Paisley, Scotland). 2,000 viable cells/well were seeded in Microtest II plates (Falcon Plastics, Oxnard, U.S.A.) in 0·1 ml medium supplemented with 1% non-essential amino acids (Eurobio, Paris, France) (test medium). Spleen cells were added 6 h later in 0·1 ml test medium at 2 effector: target cell (E:T) ratios (100:1 and 200:1). After 35–40h incubation, medium was removed from each well and plates were washed ×3 by soaking into PBS (made 5% FCS) at 37°C, emptied and left to dry. Then plates were sprayed with a protective lacquer (Condor, Milan, Italy) and the bottoms of the wells were punched out with a hand-operated cam punch press (kindly manufactured by Mr O. Schiassi, Inst. Gen. Pathology, Bologna) and transferred to scintillation vials. Lumasolve 0·1 ml (Lumac, Basle, Switzerland) was added to each vial at room temperature and, after a 30-min solubilization period, 5 ml scintillation liquid (0·4% PPO and 0·005% POPOP in toluene) were added. Vials were kept at 4°C in the dark for at least 24 h before counting in an Intertechnique SL 32 (Plaisir, France) liquid scintillation spectrometer provided with $^{226}$Ra external standard (efficiency ~40%).

Each variable was tested in sextuplicate. The results are expressed as percent cytotoxicity calculated by the formula 100(1−A/B) in which A = target cell d/min remaining after incubation with sensitized spleen cells, and B = target cell d/min remaining after incubation with unsensitized spleen cells. Spontaneous cytotoxicity was calculated as 100(1−B/C) in which C = target cell d/min remaining after incubation in the absence of spleen cells. Target cells used as specificity control in the cytotoxic anti-SV40 TASA response were the following: BALB/3T3 (normal fibroblasts) and M-MSV-BALB/3T3 (non-producer MMSV-transformed BALB/c 3T3), obtained from The American Type Culture Collection, Rockville, Md, U.S.A.

The Winn neutralization assay was performed by the method described by Glaser & Lotan (1979): 9–10-week-old BALB/c females were immunized s.c. with $10^{6}$ KD2SV, KB/cSV or mKSA-Tu5 cells. Twenty days later, spleen cells from 2–3 animals were pooled, mixed with $10^{5}$ freshly harvested mKSA(ASC) cells at different ratios (25:1, 50:1, 100:1), suspended in 0·2 ml PBS and injected s.c. (left hind leg) into 11–12-week-old BALB/c females (9–10 animals per group). Statistical evaluation was performed by $t$ and factorial $\chi^2$ tests.

The results from the *in vitro* cytotoxicity test are shown in Fig. 1. The immunization with KD2SV cells did not induce a cytotoxic response against syngeneic SV40-transformed cells higher than that after immunization with syngeneic cells;

FIG. 1.—*In vitro* cytotoxicity (%) of spleen cells from BALB/c mice immunized with KB/cSV (□) or KD2SV (■) cells against KB/cSV target cells. Mean ± s.e. of 5 experiments. Spontaneous cytotoxicity by unsensitized spleen cells was: 17 ± 6 at E:T 100:1 and 27 ± 10 at E:T 200:1.

FIG. 2.—Tumour-free female BALB/c mice (%) in the Winn neutralization assay. Each animal received a single s.c. injection of $10^5$ mKSA(ASC) cells mixed with spleen cells from BALB/c females immunized with KB/cSV (□), KD2SV (▨) or mKSA-Tu5 (▦) cells, or unimmunized (■). In the combination KB/cSV, 25:1, it was possible to evaluate only 7 of the 9–10 animals usually used in the other groups. Evaluation was performed after the death of all tumour-bearing animals, *i.e.* 4 months after mKSA(ASC) treatment.

either way there was little cytotoxicity. We also immunized female mice with cells subjected to $\gamma$-irradiation (50 Gy); again no adjuvant effect was found. The same result was found when male BALB/c mice were immunized with the same cell lines.

Neither the Winn neutralization assay (Fig. 2) nor the *in vitro* cytotoxicity test detected an adjuvant effect by minor histocompatibility antigens. Strong protection is observed (Winn assay) by syngeneically immunized spleen cells, even though mKSA-Tu5 cells are less (though not significantly) effective than KB/cSV cells. The protection induced by KD2SV cells is never greater than that due to either of the 2 syngeneic lines, being statistically similar to that with mKSA-Tu5 and lower than that with KB/cSV.

Using an *in vitro* cytotoxicity test involving cell prelabelling with $^3$H-proline, we found a low response against syngeneic SV40-transformed cells in a H-$2^d$ haplotype strain, compared to the higher amount found in a H-$2^b$ strain (Table). In fact, C57BL/6 mice immunized with a syngeneic SV40-transformed cell line (C57SV,

TABLE.—*Anti-SV*40 *TASA response in BALB/c and C*57*BL*/6 *mice evaluated by the* $^3$H-*proline cytotoxicity test* (*mean* ± *s.e.*)

| Effector | Immunizing cell | Target cell | % cytotoxicity at E:T 100:1 | 200:1 |
|---|---|---|---|---|
| BALB/c (5)* | KB/cSV | KB/cSV | 14 ± 4 | 24 ± 8 |
| C57BL/6 (4) | C57SV | C57SV | 68 ± 4 | 73 ± 1 |

* No. of experiments.

from Dr G. Trinchieri) showed strong cytotoxicity against the immunizing cells. The same phenomenon was detected by Knowles *et al.* (1979), who used a different assay ($^{51}$Cr release).

The low-responding BALB/c system could be a suitable model for detecting enhancement of *in vitro* cytotoxicity. Nevertheless, the results of *in vitro* cytotoxicity do not support the hypothesis of an adjuvant effect by minor histocompatibility antigens on the response to SV40-induced TASA, and are further confirmed by the Winn test results. We wish to underline that, with the latter technique, the spleen cells sensitized against syngeneic cells are able to neutralize almost completely the growth of a syngeneic H-$2^d$ tumour.

In conclusion, both the $^3$H-proline *in vitro* cytotoxicity test and the Winn neutralization assay failed to detect an enhancement of the immune response against SV40-transformed syngeneic cells in BALB/c mice immunized with H-2 matched SV40-transformed cells differing at multiple minor histocompatibility loci.

This work was supported by Grant 80.01564.96 of Progetto Finalizzato "Controllo della Crescita Neoplastica" from CNR, Italy.

C. D. Giovanni is in receipt of a Fellowship of Lega Italiana per la Lotta contro i Tumori, Roma, and P. L. Lollini in receipt of a Fellowship of Fondazione Anna Villa Rusconi, Varese, Italy.

### REFERENCES

BOONE, C. W., PARANJPE, M., ORME, T. & GILLETTE, R. (1974) Virus-augmented tumor transplantation antigens: Evidence for a helper antigen mechanism. *Int. J. Cancer*, **13**, 543.

COLNAGHI, M. I. (1975) Histocompatibility antigens acting as helper determinants for tumor-associated antigens of murine lymphosarcoma. *Eur. J. Immunol.*, **5**, 241.

COLNAGHI, M. I., MENARD, S. & PIEROTTI, M. A. (1978) *In vivo* resistance and *in vitro* cellular reactivity against lymphosarcoma cells in immunized mice. *Tumori*, **64**, 371.

DI MARCO, A. T., FRANCESCHI, C. & PRODI, G. (1972) Helper activity of histocompatibility antigens on cell-mediated immunity. *Eur. J. Immunol.*, **2**, 240.

DRAPKIN, M. S., APPELLA, E. & LAW, L. W. (1974) Immunogenic properties of soluble tumor-specific transplantation antigen induced by Simian Virus 40. *J. Natl Cancer Inst.*, **52**, 259.

GALILI, N., NAOR, B., ÅSIÖ, B. & KLEIN, G. (1976) Induction of immune responsiveness in a genetically low-responsive tumor–host combination by chemical modification of the immunogen. *Eur. J. Immunol.*, **6**, 473.

GLASER, M. & LOTAN, R. (1979) Augmentation of specific tumor immunity against a syngeneic SV40-induced sarcoma in mice by retinoic acid. *Cell. Immunol.*, **45**, 175.

KNOWLES, B. B., KONCAR, M., PFIZENMAIER, K., SOLTER, D., ADEN, D. P. & TRINCHIERI, G. (1979) Genetic control of the cytotoxic T cell response to SV40 tumor-associated specific antigen. *J. Immunol.*, **122**, 1798.

LAKE, P. & DOUGLAS, T. C. (1978) Recognition and genetic control of helper determinants for cell surface antigen Thy-1. *Nature*, **275**, 220.

MARTIN, W. J., WUNDERLICH, J. R., FLETCHER, F. & INMAN, J. K. (1971) Enhanced immunogenicity of chemically-coated syngeneic tumor cells. *Proc. Natl Acad. Sci. U.S.A.*, **68**, 469.

MÜLLBACHER, A. & BRENAN, M. (1980) Cytotoxic T-cell response to H-Y in "non-responder" CBA mice. *Nature*, **285**, 34.

SHEARER, G. M. (1974) Cell-mediated cytotoxicity to trinitrophenyl-modified syngeneic lymphocytes. *Eur. J. Immunol.*, **4**, 527.

STREILEIN, J. W. & STEIN-STREILEIN, J. (1978) Allogeneic reactions, "adjuvant effect" and response to weak transplantation antigens in hamsters. *Fed. Proc.*, **37**, 2052.

STUTMAN, O., SHEN, F.-W. & BOYSE, E. (1977) Ly phenotype of T cells cytotoxic for syngeneic mouse mammary tumors: Evidence for T cell interactions. *Proc. Natl Acad. Sci. U.S.A.*, **74**, 5667.

TING, C.-C. & LAW, L. W. (1977) Studies of H-2 restriction in cell-mediated cytotoxicity and transplantation immunity to leukemia-associated antigens. *J. Immunol.*, **118**, 1259.

TRINCHIERI, G., ADEN, D. P. & KNOWLES, B. B. (1976) Cell-mediated cytotoxicity to SV40-specific tumour-associated antigens. *Nature*, **261**, 312.